# Challenges Facing the Detection of Colonic Polyps: What Can Deep Learning Do?

**DOI:** 10.3390/medicina55080473

**Published:** 2019-08-12

**Authors:** Samy A Azer

**Affiliations:** Department of Medical Education, King Saud University, College of Medicine, Riyadh 11461, Saudi Arabia; azer2000@optusnet.com.au; Tel.: +966-114-699-178

**Keywords:** deep learning, convolutional neural network (CNN), colonic polyps, colorectal cancer, adenoma, colonoscopy, artificial intelligence, computer-aided diagnosis, surveillance

## Abstract

Colorectal cancer (CRC) is one of the most common causes of cancer mortality in the world. The incidence is related to increases with age and western dietary habits. Early detection through screening by colonoscopy has been proven to effectively reduce disease-related mortality. Currently, it is generally accepted that most colorectal cancers originate from adenomas. This is known as the “adenoma–carcinoma sequence”, and several studies have shown that early detection and removal of adenomas can effectively prevent the development of colorectal cancer. The other two pathways for CRC development are the Lynch syndrome pathway and the sessile serrated pathway. The adenoma detection rate is an established indicator of a colonoscopy’s quality. A 1% increase in the adenoma detection rate has been associated with a 3% decrease in interval CRC incidence. However, several factors may affect the adenoma detection rate during a colonoscopy, and techniques to address these factors have been thoroughly discussed in the literature. Interestingly, despite the use of these techniques in colonoscopy training programs and the introduction of quality measures in colonoscopy, the adenoma detection rate varies widely. Considering these limitations, initiatives that use deep learning, particularly convolutional neural networks (CNNs), to detect cancerous lesions and colonic polyps have been introduced. The CNN architecture seems to offer several advantages in this field, including polyp classification, detection, and segmentation, polyp tracking, and an increase in the rate of accurate diagnosis. Given the challenges in the detection of colon cancer affecting the ascending (proximal) colon, which is more common in women aged over 65 years old and is responsible for the higher mortality of these patients, one of the questions that remains to be answered is whether CNNs can help to maximize the CRC detection rate in proximal versus distal colon in relation to a gender distribution. This review discusses the current challenges facing CRC screening and training programs, quality measures in colonoscopy, and the role of CNNs in increasing the detection rate of colonic polyps and early cancerous lesions.

## 1. Introduction

Colorectal cancer (CRC) is responsible for approximately 10% of cancer-related mortality in western countries and is considered the second leading cause of death from cancers in the United States and the United Kingdom [1]. Approximately more than half of these cancers occur in developed countries. However, there are significant geographical variations in the incidence and mortality of CRC, with the highest estimated rates in the Australia and New Zealand region (age-standardized incidence rates of 45 per 100,000 and 32 per 100,000 in males and females, respectively), and the lowest estimated rates in western Africa (approximately 4 per 100,000) [1,2]. Several risk factors have contributed to the progressive increase in incidence of CRC, including advancing age, western dietary habits, smoking, a sedentary lifestyle, and obesity [3]. Those with long-standing inflammatory bowel disease, a family history of CRC, familial adenomatous polyposis, or hereditary non-polyposis colon cancer (HNPCC) have a greater risk of developing CRC. A personal history of colorectal adenomatous polyps is another important risk factor. Adenomas of the colon are a precursor to CRC in the majority of cases. Adenomas are estimated to be present in 20–53% of the U.S. population older than 50 years of age, and adults have approximately a 5% lifetime risk of developing adenocarcinomas [4].

Colonic adenomas may be flat, sessile, sub-pedunculated, or pedunculated. According to current opinion, CRC can develop via three different pathways [5]. First, approximately 50–75% of all CRCs evolved from conventional adenomas through chromosomal instability pathways or microsatellite instability pathways, resulting in gene mutations in a process referred to as the adenoma-to-carcinoma sequence. At the molecular level, these changes involve the Wnt pathway, TP53, KRAS, and BRAF mutations as the underlying pathophysiological mechanisms. In the absence of signal transduction pathways such as Wnt, the binding of the APC tumor suppressor protein to beta-catenin produces a destruction complex [6,7], and the formation of phosphorylation of beta-catenin by GSK3β, resulting finally in degradation into the proteasome [6]. A cascade of events take place, resulting in the disruption of the APC/axin 1/GSK3β complex and cytoplasmic accumulation of unphosphorylated beta-catenin [6,8]. The changes downstream are related to cell-cycle regulation or apoptosis. More recently, attention has been paid to microsatellite instability. A fraction develops cancerous changes through an alternative hypermutation pathway, resulting in the alteration of protein products and the development of high-frequency microsatellite instability. The second pathway is the “Lynch syndrome” mutator route (3–5%). While Lynch syndrome has historically been referred to as a “non-polyposis” syndrome, a subset of patients actually present with an attenuated polyposis-like phenotype [9]. The third pathway involves the sessile serrated pathway (15–20%). Sessile serrated polyps are usually found in the ascending (proximal) colon. These lesions are heterogeneous, yet can be differentiated from conventional adenomas. They have saw-toothed and luminal serrations with dilatations in the bases of the colonic crypts. Serrated polyps can be classified into three different types: hyperplastic polyps, sessile serrated adenomas/polyps, and traditional serrated adenomas. The latter two types of lesions are associated with CRC development [10]. Factors that increase their risk of being cancerous are an increase in the polyp size, advanced age, a history of smoking, a family history of cancer, and non-use of nonsteroidal anti-inflammatory drugs (NSAIDs) [11]. Interestingly, two studies reported a significant reduction in colorectal cancers in the left colon as compared to the right colon. The differences in mortality could be related to a significant proportion of missed adenomas/polyps, particularly sessile serrated polyps, during primary colonoscopy [12,13].

This review focuses on colonic polyps (adenomatous polyps) and the factors that increase their risk of becoming cancerous, the significance of screening for CRC, colonoscopy training programs, quality measures in colonoscopy, the current opinion on the role of convolutional neural networks (CNNs) in the early detection of colonic polyps and cancerous lesions, and future research directions in this field.

## 2. CRC Screening and Surveillance

Colonoscopy is the cornerstone of CRC screening programs. It can enable the early detection of cancerous lesions or be used as a follow-up to another screening test. Adhering to the nationally recognized guidelines for determining the interval between colonoscopies helps to minimize the development of cancer [14]. However, the challenge we currently face is that of “interval CRCs”, which have been defined as “colorectal cancer diagnosed after a screening or surveillance exam in which no cancer is detected, and before the date of the next recommended exam”. These cancers are believed to arise from serrated polyps and have mostly been found in the proximal colon [15,16]. They account for 3.4–9% of all cases of CRC. The possible explanation for an “interval CRC” may include (1) the predecessor polyp being too small on initial examination and thus challenging to detect, (2) the patient being inadequately prepared for the colonoscopy, (3) the cecum being incompletely visualized (the failure of colonoscopists to routinely intubate the cecum is a known reason limiting colonoscopy outcomes and effectiveness), (4) difficulty in pathological interpretation, and (5) the polyps being inadequately resected [17]. Studies using tandem colonoscopy (studies in which patients undergo a colonoscopy twice in the same day) have provided strong evidence that colonoscopists miss small colorectal polyps and even larger polyps [18]. In Hixson et al., a prospective tandem colonoscopy study was performed by two alternating examiners to determine the proportion of polyps missed during a colonoscopy examination. The study comprised 90 patients, and three groups were identified on the basis of lesion size. In the first group, a total of 58 lesions were detected in 31 patients, where no neoplastic lesion greater than or equal to 10 mm in size was missed. In the second group, 16% of lesions/neoplastic polyps less than or equal to 5 mm in size were missed by the first examiner. In the third group, 12.3% of the medium-sized (6–9 mm) neoplastic polyps were missed by the first examiner. The authors concluded that even an experienced colonoscopist is likely to miss approximately 15% of the colorectal neoplastic polyps that are less than 10 mm in size in the setting of adequate bowel preparation. However, polyps greater than or equal to 10 mm were rarely missed [19]. The study showed that a colonoscopy is a highly operator-dependent procedure with respect to the detection of colonic polyps/lesions. Accumulated data since then suggest that endoscopist-related factors are responsible in over 75% of interval cancers, including missed lesions or incompletely resected lesions [20,21]. These concerns have triggered an emphasis on improving the quality of colonoscopies and training programs in this area, and hence reducing CRC incidence.

## 3. Quality Measures in Colonoscopy

Quality assurance in colonoscopy has emerged as a necessity over the last decade. As stated above, this is based on evidence from several studies that show that colonoscopy outcomes are operator-dependent, and that some colonoscopists miss more polyps than others. The research on quality measures for colonoscopies continues to evolve, particularly with respect to the advantages and limitations of each measure and the degree to which each measure can reduce the burden of CRC; it is recommended that each endoscopist’s colonoscopy performance should be tested regularly. There is general agreement that three main measures warrant consideration.

The first measure is the adenoma detection rate (ADR), which is defined as the proportion of screening colonoscopies where at least one adenoma is detected. The current ADR benchmarks are 20–25% or higher for men and 15% or higher for women [22]. This measure is considered to be the most reliable and practical surrogate quality metric [22,23,24]. The significance of ADR has been demonstrated; for example, a patient scoped by a colonoscopist with an ADR of <20% was found to have a 10 times higher risk of post-colonoscopy cancer than when scoped by a colonoscopist with an ADR of >20% [25,26]. Interestingly, the ADR significantly increases when gastroenterologists are aware that they are being monitored, which highlights the significance of effective mentoring in training programs.

The second measure is the cecal intubation rate. This is a quality metric that is used to ensure that a colonoscopist has the ability to perform a complete examination of the cecum in almost all procedures. For the current benchmark, colonoscopists must demonstrate the ability to intubate the cecum in 90% or more of all examined cases. Interestingly, patients who underwent an examination by colonoscopists whose cecal intubation rates were 95% or higher were found to be less likely to have interval cancers, compared with patients who underwent an examination by colonoscopists whose intubation rates were less than 80%. Therefore, evaluating the cecal intubation rate is another important metric in assessing colonoscopy quality [27,28].

The third measure is the withdrawal time (WT). This metric is used to evaluate whether a colonoscopist has spent sufficient time to perform a thorough mucosal examination between intubating the cecum and removing the colonoscope from the patient. The current benchmark for withdrawal time is 6 min or more in examinations where no biopsies or polypectomies are performed [29]. Variability in colonoscopy outcomes was associated with the examination technique used during withdrawal [30]. Another study showed that the colonoscopy WT is shorter than is recommended in unmonitored gastroenterologists. However, the WT increases when gastroenterologists are aware that they are being monitored. Therefore, the implementation of systematic monitoring and an analysis of each endoscopist’s WT records may help to increase the ADR [31]. The results of a recent study involving 31,000 colonoscopies suggest that the WT has value as a quality metric [32].

Other measures of quality in colonoscopy include the accreditation and professional registration of the center, the number of performed colonoscopies, the number of conducted polypectomies, the comfort score (defined as the percentage of colonoscopies in which the participant experiences moderate or severe discomfort according to the Gloucester Comfort Scale: expected minimum <10%), and complication rates during colonoscopy (defined as the percentage of colonoscopies performed by the colonoscopist in which complications occur up to 30 days after the procedure). These complications include perforation rates associated with the colonoscopy, polypectomy bleeding, and the polypectomy perforation rate [33,34].

These measures of colonoscopy quality have been shown to be useful in assessing colonoscopists’ performance and in planning training programs to individualize feedback depending on deficiencies found in the evaluation. However, there is a need for studies that assess their long-term impacts on CRC incidence and mortality. To date, these measures have not resolved difficulties in discriminating between hyperplastic polyps and adenomas using conventional white-light observations and chromoendoscopy [35]. Current research is exploring whether deep learning, particularly convolutional neural networks, can help to overcome these challenges.

## 4. Convolutional Neural Networks

Convolutional neural networks (CNNs), which form a division of deep learning architecture, were initiated by the discovery of “natural visual perception” mechanisms in animals. The story dates back to the early 1960s when David Hubel and Torsten Wiesel made a breakthrough discovery in the visual system, the visual cortex, and visual processing at Harvard University. Their discovery triggered much research in the area that earned them the Nobel Prize in Physiology in 1980 [36]. Their finding and the recording of electrical activity in individual neurons in the brains of cats inspired further research, including that of Kunihiko Fukushima in 1980 and LeCun et al. in 1990. The work of Fukushima at the Nippon Hoso Kyokai (NHK) Broadcasting Science Research Laboratories, Tokyo, Japan, led to the proposal of “neocognition”, a mechanistic self-organizing neural network model for pattern recognition [37]. Yan LeCun et al. outlined the CNN framework [38]. Their innovation was developed further by introducing an artificial neural network system comprising multi-layers, called LeNet-5.

Approximately 16 years later, several studies explored these ideas and several methods were established to solve challenges in training deep learning CNNs. For example, the work of Krizhevsky et al. at the University of Toronto, Canada, produced a significant improvement in image classification through the introduction of AlexNet [39]. Further research enabled the development of ZfNet, VGGNet, GoogLeNet, and ResNet [40].

ResNet has been shown to have approximately 20 times the deep learning capability of AlexNet, and 8 times that of VGGNet [41]. Despite these differences, the basic components of a CNN are similar. For example, LeNet-5 comprises three levels of layers, namely the convolutional layer, the pooling layer, and the fully connected layer.

Over the last six to seven years, there has been significant improvement in CNNs. The current applications of CNNs in the detection of colonic polyps and other medical images can be summarized as follows:

1. Image classification: AlexNet was the first CNN architecture that was capable of image classification. The classification methods are based on sharing information using a hierarchy of classes. Some methods are based on the decomposition of a task into a series of steps, which led gradually into fine category classifiers and further processing. This architecture is known as the coarse-to-fine classification model. Other methods are based on subcategory classification. For example, a CNN architecture could provide a system for colorectal polyp grouping into subcategories that increases the rate of correct diagnosis and improves the quality of colonoscopic examinations. A study by Komeda et al. showed that the accuracy of the 10-fold cross-validation was 0.751, where the accuracy is measured as the ratio of the number of correct answers to the total number of answers produced by the CNN. This means that the decisions made by the CNN were correct in 7 of 10 cases [42]. Studies on use of CNN models for the automatic detection, segmentation, and histological examination of colonic polyps [42,43,44,45,46,47,48,49,50,51,52,53,54] are summarized in Table 1.
2. Object detection: This involves the use of CNNs in the detection and localization of objects. The methods that are currently used are based on obtaining generic measurements to test whether a sampled window is a potential object or not, and further pass the proposed output object to a more sophisticated detector to differentiate between a specific object and the surrounding background. This method constitutes the basis of the use of CNNs for automatic polyp detection and localization [44,45,46,47]. This involves finding the exact position of a polyp within an image, despite challenges in terms of size, shape, texture, and color, in addition to challenges with the camera’s viewpoint, light conditions, and reflection, as well as other significant obstacles for polyp localization during a colonoscopy. The accuracy provided by a CNN in this regard could help to reduce the polyp miss-detection rate and improve the diagnostic accuracy and quality of colonoscopies.
3. Object tracking: This function relies on how robust the representation of the target’s appearance is against challenges, such as viewpoint changes, illumination changes, or occlusions. The CNN architecture is built to discriminate object patches from their surrounding background using all of the available low-level cues. Consequently, the CNN architecture is able to classify image frames, including distinguishing polyp parts from normal non-polyp image frames. Thus, it can be used to improve a colonoscopy’s diagnostic performance [48,54].
4. Visual saliency detection: This technique aims to localize important regions or cues in an image. This function could help to identify specific changes that could confirm a clinical diagnosis. Another related feature is known as sparse representation, which comprises the ability to perform sparse representation tasks (improving image quality by producing new versions, including image denoising, super-resolution, and compressive sensing). These functions are essential to increasing the accuracy and discriminatory capabilities of the CNN architecture. An example is the reconstruction of images and the ultra-magnification of a tissue type and nuclear features followed by a machine-learning analysis and segmentation [49,50,52,53].

## 5. Clinical Applications of CNNs in Colonic Polyps

Table 1 summarizes the role of convolutional neural networks (CNNs) in polyp classification, localization, and detection. Thirteen studies were identified. These studies were created, as per the first author, by universities from the United States [44,51], Norway [45,54], Austria [43], Bangladesh [46], China [53], Denmark [48], France [49], Hong Kong [47], Japan [42], Switzerland [50], and the United Kingdom [52]. All studies used a convolutional neural network and were published in the year 2016 (*n* = 2), 2017 (*n* = 6), 2018 (*n* = 3), and 2019 (*n* = 2). The clinical applications that use a CNN for the early detection of CRC can be grouped into the following: (i) identification and classification of colonic polyps [42,43,44,45,46,47,48,54], (ii) prediction of tissue type and sequestration of glands [49], (iii) differentiation between benign and malignant lesions [50], (iv) histological classification of histopathological images of CRC by a CNN [52], (v) segmentation of CRC [53], and (vi) topography reconstruction of colonic mucosa from CNN images [51]. The CNNs that were used in the studies included the VGG-VD, the CNN-F (fast CNN), the CNN-M (medium CNN), the CNN-S (slow CNN), AlexNet, and GoogLeNet—one of the largest and most complex architectures. A study by Ribero et al. [43] showed that the features of “off-the-shelf” CNNs may be well-suited to the automatic classification of colonic polyps, even with a limited amount of data. These findings in relation to the classification of colonic polyps have been confirmed by other studies [42,44,45,46,47,48,54]. The sensitivity and accuracy of CNNs in the studies varied. One CNN identified polyps with an area under the receiver operating characteristic curve of 0.991, with an accuracy of 96.4% and false positive results at the rate of 7% [44]. Another study compared a visual inspection by an endoscopist and the use of a CNN, and showed that the precision was similar (87.3% vs. 86.4%, respectively), but the recall rate when the CNN was used was higher (87.6% vs. 77.0%, respectively), and the accuracy rate was higher with the CNN (85.9% vs. 74.3%, respectively) [47]. The study by Blanes-Vidal et al. compared the results from a new algorithm with those from previously reported methods and showed an accuracy of >96%, a sensitivity of 97%, and a specificity of 93% [48]. The study by Haj-Hassan et al. presented a method with an accuracy of 99.2% and results that showed that the method outperformed existing approaches based on traditional feature extraction and classification techniques [49]. The segmentation performance and tissue classification in the study by Kainz et al. were 98% and 95%, respectively [50]. Table 1 summarizes the findings on accuracy in other studies.

Recent reports, such as Kim et al. in 2015, have shown that women aged over 65 years have higher colon cancer mortality rates as compared to age-matched men, and that these women usually have aggressive cancer that affects the right (proximal) colon. Several factors may be responsible for the higher rates of proximal colon cancer in women. One of these is socio-cultural barriers that prevent women from enrolling in screening programs and receiving an early diagnosis. A second factor could be related to the higher incidence of colon cancer in the proximal colon because of difficulties with detection and the higher rates of missed lesions during colonoscopies. A third factor could be related to the fact that individuals with cancer of the proximal colon, particularly the cecum, are less likely to present with bleeding from the rectum or abdominal pain, thus making them unlikely to seek medical attention until the late stages of the disease [55]. With these challenges in mind, it is important to explore strategies that enhance screening for cancer affecting the right colon. It appears that the use of a CNN, together with a colonoscopy, may increase detection rates.

## 6. Future Directions

More work is required to improve the accuracy, sensitivity, and specificity of results obtained from CNN models for the detection and classification of colorectal polyps. The next steps should also address the following fundamental questions:

First, how much can the use of CNN architecture improve colonoscopy outcomes and quality metrices? It is expected that we will obtain an appropriate answer to this question over the next few years. In the meantime, we can improve the CNN architecture and compare the results obtained using CNN models with results obtained using other techniques developed in artificial intelligence that could help colonoscopists to enhance their diagnostic abilities and make better clinical decisions.

Second, will the use of a CNN architecture in colonoscopy help to reduce the rates of proximal CRC, which have currently not been reduced compared to distal CRC? This is a challenging area, and we hope to reduce these rates through the use of CNN technology. Studies that focus on examining this question should be given priority.

Third, will the use of a CNN architecture with colonoscopy procedures help to reduce CRC incidence and/or mortality? This is a central question that should be answered. We also need to assess the impact that an integration of a CNN and a colonoscopy has on reducing complications/limitations associated with the use of a colonoscopy only.

## 7. Conclusions

The use of a CNN architecture along with colonoscopy procedures is expected to improve colonoscopists’ performance, diagnostic accuracy and skills in polyp detection, classification, and segregation. These changes could result in a higher ADR and ultimately reduce CRC incidence and mortality. Although the use of CNNs over the last seven to eight years has shown promise, particularly with respect to enhancing the diagnostic capabilities of colonoscopists, the reported sensitivities, specificities, and accuracies of the CNNs in the literature vary significantly. Therefore, validation of reported results in a large multicenter trial is needed to improve the efficacy and specificity of CNN systems. Therefore, the use of CNNs seems to have had a significant impact in colonoscopy practice and gastrointestinal training programs. Considering the reported challenges with the detection of aggressive cancers affecting the ascending (proximal) colon in females, we believe that the use of CNNs to enhance the CRC detection rates could help to resolve this issue and possibly reduce the CRC mortality in these patients.

## Figures and Tables

**Table 1:** Summary of research assessing the role of convolutional neural networks (CNNs) in polyp classification, localization, and detection.

Author (Year) *	Study Goal/Research Question	Method Used/ Dataset Links/Other Links	Main Findings	Accuracy Method Used	University. Institute, City (Country) **
Komeda et al. (2017) [42]	Can a computer-aided diagnosis (CAD) based on CNN enable classification and diagnosis of colonic polyps?	Convolutional neural network (CNN) with a computer-aided diagnosis (CAD) system and artificial intelligence used to study endoscopic images.	The decision by the CNN was correct in 7 of 10 cases.	The system may be useful for rapid diagnosis of colorectal polyp classification. Further studies are needed to confirm the effectiveness of a CNN-CAD system in routine colonoscopy.	Kindai University Faculty of Medicine, Osaka-Sayama (Japan).
Ribeiro et al. (2016) [43]	Explore databases’ deep learning for the automated classification of colonic polyps.	Distinct architectures “off-the-shelf”, pretrained CNNs tested on an 8-HD-endoscopic image. 1200 images extracted from colonoscopy videos.	CNNs trained from scratch can be highly relevant for automated classification of colonic polyps.	Not tested. The results were compared with some commonly used features of colonic polyp classifications.	University of Salzburg, Salzburg, (Austria).
Urban et al. (2018) [44]	Test the ability of computer-assisted image analysis with CNN to improve polyp detection.	CNN tested 20 colonoscopy videos, a total of 5 h.S.A.AS Video available at: http://www.igb.uci.edu/colonoscopy/AI_for_GI.html Supplementarymaterial at https://doi.org/10.1053/j.gastro.2018.06.037.	Four expert reviewers of the videos identified eight additional polyps without CNN assistance that had not been removed and identified, and an additional 17 polyps with CNN assistance.	The CNN identified polyps with an area under the receiver operating characteristic curve of 0.991 and an accuracy of 96.4%. The CNN had a false positive rate of 7%.	University of California, California (The United States).
Qadir et al. (2019) [45]	Improve the overall performance of CNN-based polyp detection on colonoscopy images.	The method comprises two stages: a region of interest proposed by CNN detector, and a false positive reduction unit.	The bidirectional temporal information in the system design helped in estimating polyp positions and predicting false positives (improved sensitivity and precision).	Specificity improved compared to convolutional false positive learning methods.	University of Oslo, Oslo (Norway).
Billah et al. (2017) [46]	Can an automated system support in gastrointestinal polyp detection?	CNN combined with a linear support vector machine (SVM). Most of the data were collected from the Department of Electronics, University of Alcala, at http://www.depeca.uah.es/colonoscopy-dataset/. Also, Endoscopic Vision Challenge at http://polyp.grand-challenge.org.databases.	The computer-aided polyp detection reduced the rate of missing polyps and assisted in finding colonic regions to pay attention to.	The proposed system outperforms the state-of-the-art methods, gaining accuracy of 98.6%, sensitivity of 98.8%, and specificity of 98.5%.	Mawlana Bhashani Science & Technology University, Tangall (Bangladesh).
Zhang et al. (2017) [47]	Developing a fully automatic algorithm to detect and classify hyperplastic and adenomatous colorectal polyps.	A novel transfer learning application is proposed utilizing features learned from big non-medical data sets with 1.4–2.5 million images using deep convolutional neural network.	The method identified polyp images from non-polyp images in the beginning, followed by predicting the polyp histology. Automated algorithms can assist endoscopists in identifying polyps that are adenomatous but have been incorrectly judged as hyperplasia.	Compared with visual inspection by endoscopists, the results of the study show that the method has similar precision (87.3% versus 86.4%), but a higher recall rate (87.6% versus 77.0%), and a higher accuracy (85.9% versus 74.3%).	The Chinese University of Hong Kong, Shatin (Hong Kong).
Blanes-Vidal et al. (2019) [48]	Examine two proposed innovative science algorithms to improve acquisition and analysis of data obtained from capsule endoscopy on colorectal polyps	Data from the Danish National Screening Program colorectal capsule endoscopy (CCE) and colonoscopy and histopathology of all polyps were used. The algorithm system developed matched CCE and colonoscopy polyps. The deep CNN enabled autonomous detection and localization of polyps.	The system was able to objectively quantify similarities between CCE and colonoscopy polyps.	Compared to previous methods, the new algorithm showed an accuracy >96%, sensitivity of 97%, and specificity of 93%.	University of Southern Denmark, Odense, (Denmark).
Haj-Hassan et al. (2017) [49]	Can CNN predict tissue types related to colorectal cancer progression?	CNN and multispectral biopsy images of 30 patients with colorectal cancer images at three different histopathological stages.	CNN has demonstrated the ability to detect colorectal cancer types of tissues with accuracy.	The accuracy was 99.2%, outperforming existing approaches based on traditional features extraction and classification techniques.	University of Lorraine, Metz, Lorraine (France).
Kainz et al. (2017) [50]	Assess the ability of deep learning for segmentation of glands and classification to differentiate between benign and malignant tissues of the colon.	Deep neural-based approach designed for segmentation and classification of glands in colonic tissues into benign or malignant. Dataset publicly available at http://www.warwick.ac.uk/bialab/GlasContest. Trained models of the deep convolution net available at http://github.com/pkainz/glandsegmentation-models. Input data and evaluation scripts are available at http://www2.warwick.ac.uk/fac/sci/dcs/research/tia/glascontest.	The model demonstrated the ability to differentiate between benign and malignant colonic tissues with high accuracy.	The segmentation performance and tissue classification accuracy has been 98% and 95%, respectively.	University of Zurich, ETH Zurich, Zurich (Switzerland).
Mahmood and Durr (2018) [51]	Present a method using CNN-conditional random field to reconstruct topography of colonic mucosa from convolutional colonoscopy images.	The authors trained the unary and pairwise functions of conditional random field integrated in a CNN system and using data generated from endoscopic images.	The estimated depth maps can be used in reconstructing the topography of colonic mucosa.	Not tested. The system can be integrated into existing endoscopy system and the algorithm enables detection, segmentation, and classification of polyps.	Johns Hopkins University, Baltimore, MD, USA.
Sirinlukunwattana et al. (2016) [52]	Detection and classification of histopathology images of colorectal cancerous tissues among locality sensitive deep learning.	A spatiality constrained convolutional neural network (SC-CNN) was used to perform nucleus detection, and for classification a novel neighboring ensemble predictor (NEP) coupled with CNN was proposed.	A large dataset of images of colorectal adenocarcinoma cells (20,000 annotated-nuclei belonging to four different patients).	The method produced the highest average F1 score, as compared to other recently published approaches.	University of Warwick (The United Kingdom).
Men et al. (2017) [53]	Propose a novel deep dilated convolutional neural network (DD CNN)-based method for fast and consistent auto-segmentation in colorectal cancer.	Deep dilated convolutional neural network (DD CNN).	Deep dilated convolutional neural networks (DD CNN) can be used with accuracy and efficiency to contour and streamline radiotherapy.	The proposal outperformed U-Net for all segmentations. The average Dice similarity coefficient (DSC) was 3.8% higher than that of U-Net.	National Cancer Center Chinese Academy of Medical Sciences and Peking Union Medical College, Beijing (China).
Shin et al. (2016) [54]	Apply a region-based CNN architecture in automatic detection of polyps in the images obtained from colonoscopy examinations.	A deep-CNN model was used in the detection system. Image augmentation strategies were tested for training deep networks. Two post-learning methods were integrated to detect false positives and enable reliable polyp detection.	The system improved detection performance of colonic polyps.	Not measured.	Norwegian University of Science and Technology, Trondheim, (Norway)

* Only full papers published in peer-reviewed journals were included. Conference proceedings and abstracts were not included. ** The University of the first author was stated. Abbreviations: CAD = computer-aided diagnosis; CNN = convolutional neural network; DD CNN = deep dilated convolutional neural networks; DSC = Dice similarity coefficient; SVM = support vector machine (see Appendix A).

## Abbreviations

**APC**	APC Tumor suppressor protein; APC regulator of Wnt signaling pathway; (also known as BTPS2, DP2, DP3, GS, PPP1R46)
**BRAF**	B Raf proto-oncogene
**GSK3ß**	GSK3ß in interacting protein
**KRAS**	KRAS proto-oncogene; first identified in Kirsten RAT sarcoma virus
**LeNet-5**	The name reflects convolutional network of 5 layers designed for handwritten and machine-printed character recognition; created by Y. LeCun and three others
**TP53**	Tumor Protein P53
**Wnt**	Wingless NNTV integration site

## Appendix A

The Studies included in Table 1 are full research studies. No abstracts or conference proceedings. For further resources (i) Peters, T., Yang, G.Z., Navab, N., Mori, K., Luo, X., Reichl, T., McLeod. J. (Eds.). Computer-assisted and robotic endoscopy. Third International Workshop, CARE 2016, Held in Conjunction with MICCAI 2016. Athens, Greece, 17 October 2016, Springer International Publishing AG, 2017, (ii) Chen, Y.W., Tanaka, S., Howlett, R.J., Jain, L.C. (Eds.). Innovation in medicine and healthcare, 2017. Proceedings of the 5th KES interterminal conference on innovation in medicine and healthcare (KES-InMed2017). Springer International Publishing AG, 2018, (iii) Bai, W., Sanroma, G., Wu, G., Munsell, B.C., Zhan, Y., Coupe, P. (Eds.). Patch-based technologies in medical imaging. 4th international workshop, Patch-MI 2018. Held in conjunction with MICCAI 2018 Granada, Spain, 20 September 2018 Proceedings. Springer Nature, Switzerland AG, 2018.
